# Mutational Profile and Potential Molecular Therapeutic Targets of Pheochromocytoma

**DOI:** 10.3389/fendo.2022.921645

**Published:** 2022-07-28

**Authors:** Xiaosen Ma, Chao Ling, Meng Zhao, Fen Wang, Yunying Cui, Jin Wen, Zhigang Ji, Caili Zhang, Shi Chen, Anli Tong, Yuxiu Li

**Affiliations:** ^1^ Key Laboratory of Endocrinology, Department of Endocrinology, National Health Commission of the People’s Republic of China, Peking Union Medical College Hospital, Peking Union Medical College, Chinese Academy of Medical Sciences, Beijing, China; ^2^ The Laboratory of Clinical Genetics, Peking Union Medical College Hospital, Peking Union Medical College, Chinese Academy of Medical Sciences, Beijing, China; ^3^ Bioinformatics Institute, Novogene Co., Ltd., Beijing, China; ^4^ Department of Endocrinology, Tongji Hospital, Tongji Medical College, Huazhong University of Science and Technology, Wuhan, China; ^5^ Department of Urology, Peking Union Medical College Hospital, Peking Union Medical College, Chinese Academy of Medical Sciences, Beijing, China; ^6^ Department of Technical Support, Novogene Co., Ltd., Beijing, China

**Keywords:** pheochromocytoma, paraganglioma, gene, mutational profile, potential molecular therapeutic targets

## Abstract

**Purpose:**

Pheochromocytoma/paraganglioma (PCC/PGL; collectively known as PPGL) can be driven by germline and somatic mutations in susceptibility genes. We aimed to investigate the mutation profile and clinical features of pathogenic genes in highly genetically heterogeneous PPGL and to preliminary explore molecular therapeutic targets in PPGL.

**Methods:**

We established a panel of 260 genes, including susceptibility genes of PPGL and other important tumorigenic genes to sequence 107 PPGL tissues.

**Results:**

Overall, 608 genomic mutations were identified in 107 PPGL tissues. Almost 57% of PPGL tissue samples exhibited pathogenic mutations, and the most frequently mutated gene was *SDHB* (15/107, 14%). *SDHB* and *HRAS* were the most commonly mutated genes in germline-mutated PPGL (25/107, 23%) and nongermline-mutated PPGL (36/107, 34%), respectively. In addition, novel pathogenic mutations were detected in sporadic PPGL. PPGL with mutations in the hypoxia pathway had an earlier onset and higher norepinephrine level than those in the kinase pathway. Receptor tyrosine kinase (RTK; 22%, 24/107), mitogen-activated protein kinase (MAPK; 14%, 15/107), and tyrosine kinase (TK; 2%, 2/107) pathways were the most frequently mutated pathways in PPGL.

**Conclusion:**

Our results provided the genetic mutation profile in PPGL tissues. Genetic mutations in PPGL were mainly concentrated in the RTK, TK, and MAPK pathways, suggesting potential molecular therapeutic targets for PPGL.

## Introduction

Pheochromocytoma and paraganglioma (PCC/PGL; collectively known as PPGL) is a rare neuroendocrine tumor originating from chromaffin cells of the adrenal medulla or extra-adrenal paragangliomas. It represents a subset of tumors with high genetic heterogeneity. Approximately 40% cases of PPGL are caused by germline mutations in more than 20 known susceptibility genes (1). The inherent genetic mutations in PPGL fall into two major clusters in terms of their gene expression profile in tumors. Cluster I includes *VHL*, *SDHx*, *IDH*, *EPAS1*, *MDH2*, *PHD2*, and *FH* genes. Mutations in these genes cause activation of pseudo-hypoxic pathway and accumulation of hypoxia-inducible factor-1α, characteristically leading to a high expression of vasoendothelial growth factor and its receptors. Cluster II involves *RET*, *NF1*, *MAX*, and *TMEM127* genes, which are associated with abnormal activation of the kinase signaling pathway, such as the phosphatidylinositol 3-kinase (PI3K)-Akt-mechanistic target of rapamycin (mTOR) pathways (2). Although the hereditary profile of PPGL has been well characterized, the clinical and genetic features of PPGL in Chinese population are not clearly described.

Apart from the studies on germline-susceptibility-associated mutations in PPGL, some studies have examined somatic mutations in PPGL cases. Somatic mutations, including *NF1*, *RET*, *VHL*, *EPAS1*, *ATRX*, *HRAS*, *CSDE1*, *IDH1*, *BRAF*, *FGFR1*, *TP53*, *SETD2*, and *ARNT*, were reported to be related to the development of PPGL (3, 4). Castro-Vega *et al*. conducted whole-exome sequencing (WES) of 30 tumor–normal DNA pairs and identified 672 somatic variants in coding regions or at exon/intron boundaries, with few nongermline mutations in genes that were not reported previously in PPGL (4). It has been widely accepted that PPGL is mainly driven by distinct germline or somatic mutations in known susceptibility genes.

Moreover, there is still a substantial fraction of PPGL whose etiology is largely unknown. WES allows direct measurement of the status of tumor mutations. However, it remains unsuitable for screening of large quantities of samples because it is expensive, labor-intensive, and time-consuming. Most of the recurrent somatically mutated genes in PPGL were reported previously, and few novel gene mutations were identified in some studies (4–7). Therefore, the limited number of genes involved in the tumorigenesis of PPGL render it possible to establish a gene panel that is equally accurate as WES (8, 9). In this study, we established a panel of 260 genes; this included recurrently reported genes of PPGL, genes that were probably relevant for PPGL but were not reported (such as genes involved in the tricarboxylic acid cycle, pseudohypoxia, and kinase signaling pathways), and reported genes in neuroendocrine and other tumors. We sequenced the genes in 107 PPGL tissues to assess the mutational and clinical profile of PPGL and explored the possible molecular therapeutic targets of PPGL.

## Materials and Methods

### Patients and Sample Collection

A total of 107 PPGL tissue samples were collected from 107 patients who had undergone surgical resection at the Peking Union Medical College Hospital, Beijing, China. None of the patients had received chemotherapy or radiotherapy before the surgery. All the pathological diagnoses were reviewed by two pathologists. PPGL tissues were collected after obtaining informed consent from patients. Approval was obtained from the medical ethics committee of the said hospital.

### Genomic DNA Preparation and Target Sequencing

DNA was extracted from freshly frozen tumor tissues (wet weight 20–25 mg) using the QIAamp^®^Genomic DNA mini kit (QIAGEN, CA, USA) as per the manufacturer’s instructions. The probes of target regions for 260 genes (**Supplementary Table S1**) were designed against Agilent available online (http://www.agilent.com). The sequence library was generated using the Agilent SureSelectXT custom kit (Agilent Technologies, Palo Alto, CA) as per the manufacturer’s instructions. Briefly, fragmentation was performed using a hydrodynamic shearing system (Covaris, Massachusetts, USA) to generate 180–280 bp fragments. The DNA fragments were end-repaired, A-tailed, and adapter-ligated for Illumina sequencing. Further, size selection, PCR amplification, and library hybridization were performed. Each captured library with an index was loaded onto the Illumina HiSeq X platform (Illumina Inc., San Diego, CA), and 150-bp paired-end reads were generated.

### Quality Control, Detection and Filtering of Genomic Alterations

The fastq files were subjected to quality control to exclude undesirable sequences, including those with adapter contamination or low-quality or unrecognizable nucleotides. The fastq files were aligned against the Human Reference Genome (UCSC hg19) using the Burrows-Wheeler Aligner. Further, SAM (Sequence Alignment/Map) tools (10) were used to sort the BAM files and to perform duplicate marking, local realignment, and base quality recalibration to generate the final BAM file (11) for computing the sequence coverage and depth. ANNOVAR was used to annotate the Variant Call Format file obtained in the previous step (12).

The genomic mutations were screened as follows:

1) Mutations with coverage less than 10× were excluded.
2) Variants with mutant allele frequency of ≥ 0.01 in the 1,000 Genomes databases (1000g2015aug_Chinese, 1000g2015aug_eas, 1000g2015aug_all, esp6500siv2_all, ExAC_ALL, ExAC_EAS, NovoDb_WES and NovoDb_WGS) were excluded (13, 14).
3) The variants in the exonic or splicing region (10 bp upstream and downstream of splicing sites) were retained.
4) The synonymous mutations were excluded.
5) Nonsynonymous single nucleotide variants (SNVs) were retained if at least two of the functional predictions by PolyPhen-2, SIFT, MutationTaster, and CADD revealed that the SNV was pathogenic or likely pathogenic, was included in the COSMIC database, or was predicted by ACMG to be “pathogenic or likely pathogenic” (15–18).
6) “Benign” or “likely benign” variants in ClinVar were excluded.

In addition, actionable mutations, defined as variants potentially linked to drugs in registered clinical trials, were detected by searching My Cancer Genome database (https://www.mycancergenome.org).

### Mutation Validation in Blood Samples

Since susceptibility germline mutations were prevalent in patients with PPGL, we identified germline mutations in the cohort. If the mutated genes were among the 25 germline susceptibility genes (**Supplementary Table S2**), Sanger sequencing was performed to confirm whether the mutations in tumor tissues were also present in the corresponding blood samples. Accordingly, samples with known pathogenic germline mutations were detected from 107 PPGL tissues (**Supplementary Figure 1**).

### Statistical Analysis

Continuous variables with normal distribution were expressed as means ± standard deviation, and those with non-normal distribution were expressed in median (25%, 75%). Differences between two groups with parametric and nonparametric variables were assessed using Student’s *t* and Mann–Whitney *U* tests, respectively. Categorical data were reported as numbers or corresponding percentages, and two groups were compared using χ^2^ test. P < 0.05 was considered statistically significant. SPSS version 23.0 for Windows was used for all statistical analyses.

## Results

### Clinical and Sequencing Data Obtained From PPGL Tissues

A total of 107 PPGL tissues were analyzed, involving tumors from 50 male and 57 female patients, with a mean age of 40 ± 14 years (range: 6–64 years). Overall, 8 multiple PPGL, 54 PGL, and 45 PCC samples were included in our cohort. In total, 8 of 107 cases had PPGL with distant metastasis (**Table 1** and **Supplementary Table S3**).

**Table 1:** Clinical features of PPGL.

Clinical characteristics	Total (n=107)	PPGL withGermline mutation (n=25)	Sporadic PPGL (n=82)	P value
Sex (M/F)	50/57	11/14	39/43	0.755
Age (year)	44 (30,54)	27 (24,37)	45 (32,54)	<0.001^*^
Duration (month)	24 (5,60)	36 (11,84)	24 (4,48)	0.03^*^
SBP	185 (150,220)	180 (160,210)	188 (151,220)	0.707
DBP	110 (92,120)	110 (102,120)	107 (90,120)	0.25
Family History	5/107	5/25	0/82	<0.001^*^
Multiple	8/107	4/25	4/82	0.157
Location				
PCC	49/107	8/25	41/82	0.114
PGL	62/107	19/25	43/82	0.064
HN-PGL	3/107	3/25	0/82	0.012^*^
T-PGL	2/107	2/25	0/82	0.053
R-PGL	53/107	15/25	38/82	0.232
P-PGL	7/107	2/25	5/82	1
Tumor size (cm)	4.4 (3.9,7.0)	4.6 (3.1,5.8)	5.0 (3.8,6.5)	0.201
Metastasis	8/107	6/25	2/82	0.002^*^
24h NE	103 (36,373)	250.4 (126.0,534.6)	79.9 (36.2,336.8)	0.024^*^
24h E	3 (2,5)	3.8 (2.7,4.4)	4.1 (2.7,6.1)	0.318
24h DA	224 (161,329)	274.5 (171.0,453.6)	218.7 (163.2,324.2)	0.174

SBP.systolic blood pressure; DBP.diastolic blood pressure; PCC.pheochromocytoma; PGL.paraganglioma;

HN-PGL.head and neck paraganglioma; T-PGL.thoracic paraganglioma; R-PGL.retroperitoneal paraganglioma; P-PGL.pelvic paraganglioma;

NE.Norepinephrine, normal range: 16.7~40.7 μg/24 h; E.Epinephrine, normal range: 1.7~6.4 μg/24 h; DA.Dopamine, normal range: 120.9~330.6 μg/24 h.

^*^ Significant difference between PPGL with Germline mutation and Sporadic PPGL (P<0.05).

We achieved an average sequencing depth of 346× (range 265–515×) in all samples. After multiple-step filtering, 608 genomic mutations were identified in the PPGL samples, including 528 missense, 17 stop-gain, 23 splicing, and 39 indel mutations (**Supplementary Table S4**). Moreover, one synonymous SNV (*VHL*: c.A414G, p.P138P) that was reported to be pathogenic was included (19). The overall frequency was 3.8 (range 0–7) mutations per Mb in tumor tissues upon the aforementioned filtration.

### Germline Mutations and Nongermline Mutations in PPGL

**Figure 1:**
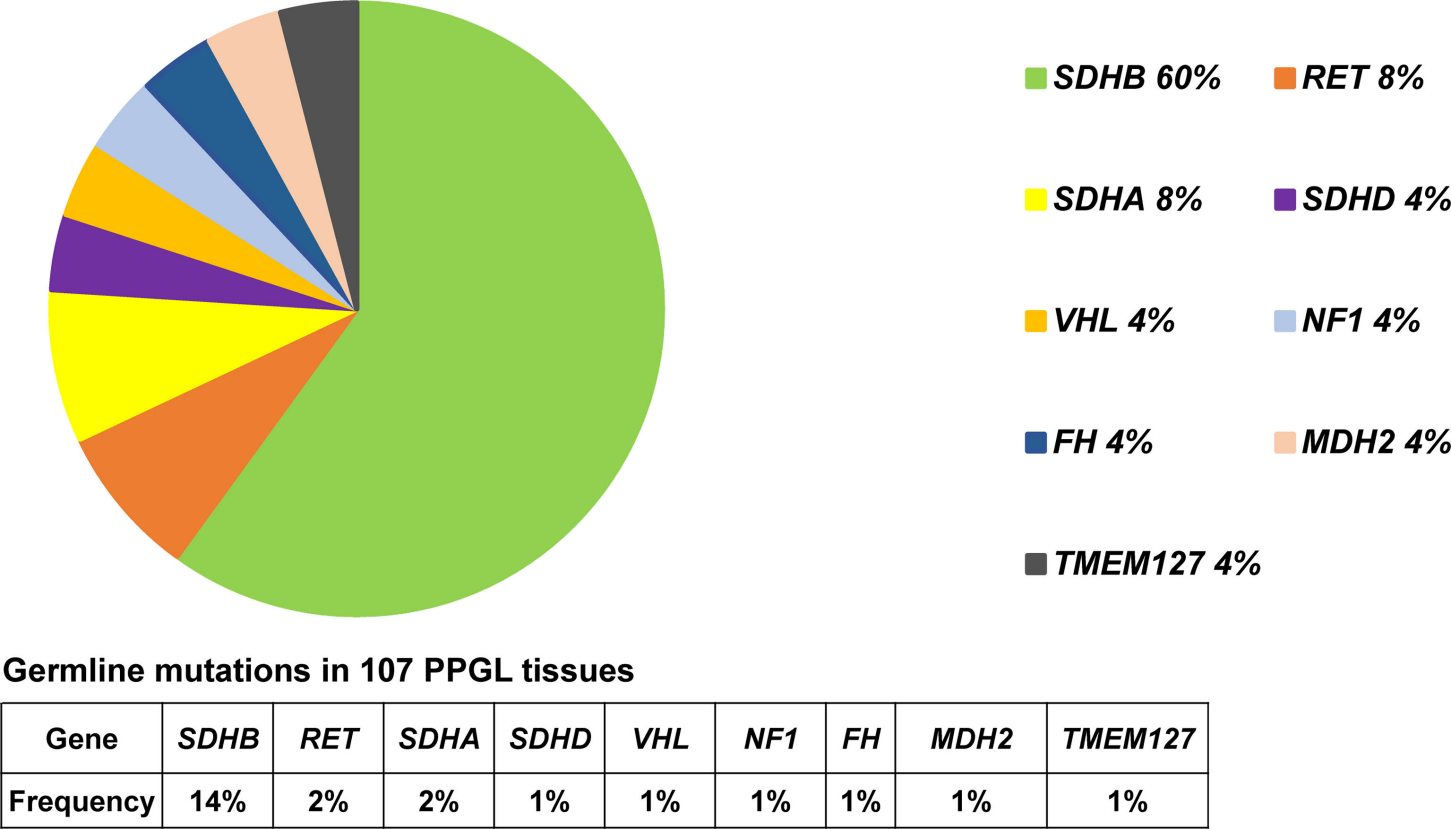
Pie diagram showing the distribution of germline mutations in PPGL. Circular statistical graphic was used to illustrate the proportion of germline mutations.

Predisposing germline mutations were identified in 25 (23%) of 107 patients. The most frequent mutations in 25 patients occurred in *SDHB* (15/25, 60%) and followed by *RET* (2/25, 8%), *SDHA* (2/25, 8%). Germline mutations in *VHL*, *SDHD*, *NF1*, *FH, MDH2*, and *TMEM127* were rare, standing at 4% (1/25) each (**Table 2** and **Figure 1**).

**Table 2:** Pathogenic mutations in PPGL.

Patients	Gene	DNA Change	Protein Change	Frequency
Germline mutations in PPGL				
P116	*SDHB*	c.C136T	p.R46X	1/107 (1%)
P79	*SDHB*	c.G137A	p.R46Q	1/107 (1%)
P108	*SDHB*	c.170delA	p.H57fs	1/107 (1%)
P76	*SDHB*	c.T277C	p.C93R	1/107 (1%)
P24	*SDHB*	c.331_332del	p.L111fs	1/107 (1%)
P119	*SDHB*	c.423+1G>A		1/107 (1%)
P60	*SDHB*	c.T574G	p.C192G	1/107 (1%)
P6/P111	*SDHB*	c.G689A	p.R230H	2/107 (2%)
P75	*SDHB*	c.G725A	p.R242H	1/107 (1%)
P63/P117	*SDHB*	c.765+1G>A		2/107 (2%)
P77/P118	*SDHB*	c.766-1G>A		2/107 (2%)
P13	*SDHB*	large deletions of exon1		1/107 (1%)
P109	*SDHD*	c.177_181del	p.S59fs	1/107 (1%)
P95	*VHL*	c.A414G	p.P138P	1/107 (1%)
P9	*SDHA*	c.C508A	p.Q170K	1/107 (1%)
P122	*SDHA*	c.G1865A	p.W622X	1/107 (1%)
P41	*FH*	c.G817A	p.A273T	1/107 (1%)
P78	*MDH2*	c.G523A	p.V175I	1/107 (1%)
P104	*RET*	c.G1891T	p.D631Y	1/107 (1%)
P128	*RET*	c.G1901A	p.C634Y	1/107 (1%)
P66	*NF1*	c.6557delA	p.E2186fs	1/107 (1%)
P93	*TMEM127*	c.T133C	p.C45R	1/107 (1%)
Non-germline known pathogenic mutations in PPGL				
P121	*EPAS1*	c.C1589T	p.A530V	1/107 (1%)
P17/P21 P103/P302	*EPAS1*	c.C1591T	p.P531S	4/107 (4%)
P50/P135	*EPAS1*	c.C1592A	p.P531H	2/107 (2%)
P11	*EPAS1*	c.C1592G	p.P531R	1/107 (1%)
P2	*EPAS1*	c.A1595G	p.Y532C	1/107 (1%)
P84	*SDHD*	c.C139T	p.Q47X	1/107 (1%)
P26	*SDHD*	c.C112T	p.R38X	1/107 (1%)
P92	*VHL*	c.G245T	p.R82L	1/107 (1%)
P141	*VHL*	c.G445A	p.A149T	1/107 (1%)
P99	*FH*	c.G206A	p.G69D	1/107 (1%)
P1/P20/ P36/P126 P127/P137	*HRAS*	c.C181A	p.Q61K	6/107 (6%)
P48/P49/P53 P124/P130/P136	*HRAS*	c.A182G	p.Q61R	6/107 (6%)
P58/P85	*RET*	c.T2753C	p.M918T	2/107 (2%)
P140	*RET*	c.C1902G	p.C634W	1/107 (1%)
P125	*NF1*	c.C6907T	p.Q2303X	1/107 (1%)
P131	*NF1*	c.4159_4171del	p.A1387fs	1/107 (1%)
P14/P22	*FGFR1*	c.C1371G	p.N457K	2/107 (2%)
P16	*H3F3A*	c.G103T	p.G35W	1/107 (1%)
P73	*BRAF*	c.A1801G	p.K601E	1/107 (1%)
P116	*TP53*	c.C833G	p.P278R	1/107 (1%)
P117	*TP53*	c.365_366del	p.V122fs	1/107 (1%)
P117	*ATRX*	c.A2854T	p.K952X	1/107 (1%)
P130	*ATRX*	c.C3518A	p.S1173X	1/107 (1%)
P27	*ATRX*	c.G3985T	p.E1329X	1/107 (1%)
P100	*SMO*	c.G1769C	p.S590T	1/107 (1%)
P114	*FGFR2*	c.G943A	p.A315T	1/107 (1%)
Novel pathogenic mutations in PPGL				
P27	*PIK3CA*	c.A3140G	p.H1047R	1/107 (1%)
P35	*PRKACA*	c.T617G	p.L206R	1/107 (1%)

Clinical features of PPGL with or without germline mutation were shown in **Table 1**. PPGL with germline mutations often exhibited a family history of PPGL and a younger age of onset and were more likely to develop metastasis.

**Figure 2:**
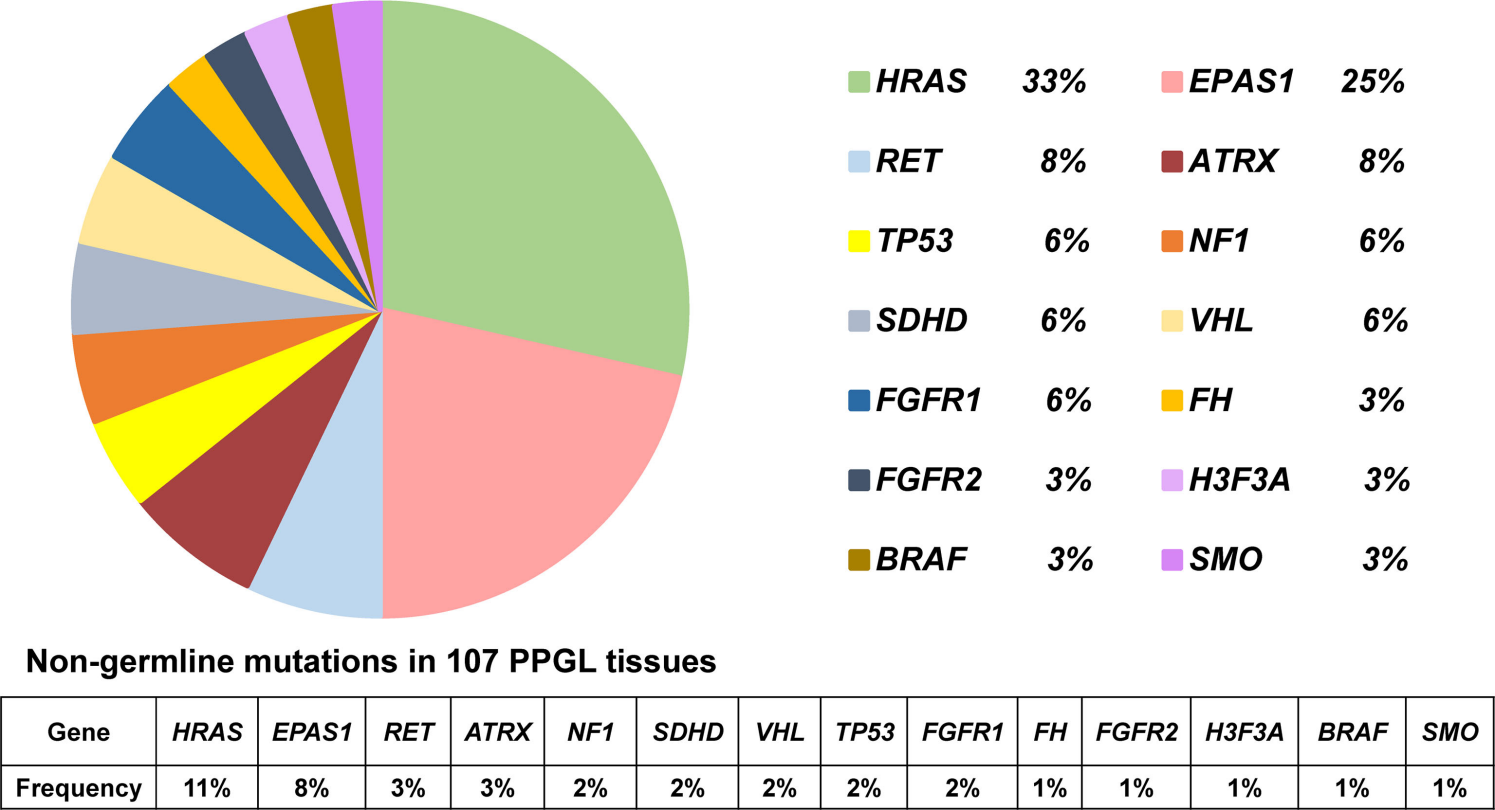
Pie diagram showing the distribution of nongermline mutations in PPGL. Circular statistical graphic was used to illustrate the proportion of nongermline mutations.

In 36 PPGL tissues without a recognizable germline mutation, 42 nongermline pathogenic variants were detected. The most frequently mutated gene was *HRAS* at a rate of 12/36 (33%), followed by *EPAS1* (9/36, 25%), *RET* (3/36, 8%), *ATRX* (3/36, 8%), *NF1* (2/36, 6%), *SDHD* (2/36, 6%), *VHL* (2/36, 6%), *FGFR1* (2/36, 6%), *TP53* (2/36, 6%), *H3F3A* (1/36, 3%), *FH* (1/36, 3%), *FGFR2* (1/36, 3%), *BRAF* (1/36, 3%), and *SMO* (1/36, 3%; **Table 2** and **Figure 2**).

It should be noted that both a pathogenic *SDHB* germline mutation and *TP53* somatic mutation were detected in PPGL lung metastases. *SDHB* germline pathogenic, *TP53* somatic, and *ATRX* somatic variants were simultaneously detected in a primary PPGL tissue, which further developed distant metastasis. In addition, both *PIK3CA* and *ATRX* somatic variants were detected in a bladder PGL tissue. *HRAS*, *ATRX*, and *BRAF* somatic variants were concurrently detected in a retroperitoneal PGL tissue.

### Novel Pathogenic Genes in PPGL

Novel pathogenic mutations such as *PIK3CA* (1/107, 1%) and *PRKACA* (1/107, 1%) were detected in two different patients with bladder PGL, respectively (**Tables 1**, **2**). Both these mutations in *PIK3CA* and *PRKACA* were verified as somatic heterozygous variants with a mutation rate of 50.5% (95/188) and 36.9% (24/65), respectively. Interestingly, both *PIK3CA* and *PRKACA* were involved in the tyrosine kinase (TK) pathway.

### Mutation Profile of PPGL With *SDHB* Germline Mutation

*SDHB* mutations were detected in 15 of 107 PPGL (**Table 2** and **Figures 1** and **2**) tissues. Overall, 6 patients with *SDHB* mutations exhibited metastasis. Among the metastatic cases, 5 and 1 sample were from primary lesions and pulmonary metastatic focus, respectively. *SDHB* germline mutations were mainly concentrated in exon 2 and exon 7, including 7 missense, 2 frameshift, 1 stop-gain, and 5 splicing mutations.

Interestingly, mutations of *TP53* (2/107, 1.9%) exclusively appeared in *SDHB*-mutated PPGL. All cases (2/107, 1.9%) with combined *SDHB* and *TP53* mutations exhibited metastatic PPGL. In addition, *ATRX*, a risk factor associated with metastatic PPGL, was detected at a rate of 2.8% (3/107). *ATRX* somatic and *SDHB* germline mutations were both detected in a case of metastatic PGL. Simultaneous mutations in *ATRX* and *PIK3CA* were detected in a case of bladder PGL. Additionally, pathogenic mutations (*ATRX*, *HRAS*, and *BRAF*) were detected in the same PGL case with high levels of 24-h urinary norepinephrine and epinephrine.

### Clinical Features of PPGL in Different Pathways

Pathogenic mutations of PPGL were clustered in the hypoxia (n = 35) and kinase signaling (n = 26) pathways. PPGL with mutations in the hypoxia pathway had an earlier onset, higher norepinephrine levels, and was more likely to develop PGL than that with mutations in the kinase signaling pathway. PPGL with mutations in the kinase signaling pathway exhibited a higher epinephrine level (**Table 3**).

**Table 3:** Comprison of clinical characteristics of PPGL in different pathways.

Clinicalcharacteristics	PPGL with mutations in the Hypoxia pathway (n=35)	PPGL with mutations in the Kinase pathway (n=26)	P Value
Sex(M/F)	14/21	16/10	0.096
Age(year)	30 (25,44)	49 (42,56)	<0.001^*^
Duration(month)	24 (9,78)	24 (4,48)	0.160
SBP	180 (160,200)	200 (162,240)	0.201
DBP	110 (100,120)	110 (100,136)	0.832
Family History	5/35	0/26	0.066
Multiple	6/35	1/26	0.228
Location			
PCC	11/35	15/26	0.021^*^
PGL	27/35	11/26	0.006^*^
HN-PGL	3/35	0/26	0.254
T-PGL	2/35	0/26	0.503
R-PGL	20/35	11/26	0.252
P-PGL	2/35	0/26	0.503
Tumor size(cm)	4.5 (2.9,6.5)	5.1 (4.0,7.2)	0.165
Metastasis	7/35	0/26	0.017^*^
24h NE	250.4 (130.5,958.0)	57.3 (33.9,183.5)	<0.001^*^
24h E	3.8 (2.7,4.4)	5.6 (3.3,25.3)	0.007^*^
24h DA	248.7 (174.7,362.2)	208.5 (149.6,328.4)	0.484

SBP.systolic blood pressure; DBP.diastolic blood pressure; PCC.pheochromocytoma; PGL.paraganglioma;

HN-PGL.head and neck paraganglioma; T-PGL.thoracic paraganglioma; R-PGL.retroperitoneal paraganglioma; P-PGL.pelvic paraganglioma;

NE.Norepinephrine, normal range: 16.7~40.7 μg/24 h; E.Epinephrine, normal range: 1.7~6.4 μg/24 h; DA.Dopamine, normal range: 120.9~330.6 μg/24 h.

*Significant difference between PPGL with mutations in the hypoxia pathway and with mutations in the kinase signaling pathway (P < 0.05).

To explore whether the differences were due to the high proportion of germline mutations in the hypoxia pathway, we compared the two pathways in nongermline-mutated PPGL. Surprisingly, the disparities in onset age and norepinephrine level persisted (**Table 4**). Therefore, we concluded that PPGL associated with the hypoxia pathway had an earlier onset and higher norepinephrine level than that with the kinase signaling pathway irrespective of germline or nongermline level. However, in the hypoxia pathway, no significant differences were detected between PPGL with germline and somatic mutation (**Table 5**).

**Table 4:** Comparison of clinical characteristics of PPGL with somatic mutations in different pathways.

Clinicalcharacteristics	PPGL with somatic mutations in the Hypoxia pathway (n=14)	PPGL with somatic mutations in the Kinase pathway (n=22)	P Value
Sex(M/F)	3/11	13/9	0.061
Age(year)	36 (26,50)	53 (47,56)	0.009^*^
Duration(month)	11 (7,24)	30 (6,48)	0.619
SBP	180 (160,198)	200 (168,240)	0.071
DBP	100 (100,110)	110 (100,155)	0.293
Family History	0/14	0/22	
Multiple	2/14	1/22	0.547
Location			
PCC	7/14	11/22	1.000
PGL	8/14	11/22	0.742
HN-PGL	0/14	0/22	
T-PGL	0/14	0/22	
R-PGL	8/14	11/22	0.676
P-PGL	0/14	0/22	
Tumor size(cm)	4.8 (3.0,6.0)	5.1 (4.0,7.5)	0.281
Metastasis	1/14	0/22	0.389
24h NE	258.5 (115.8,1052.1)	54.1 (33.7,111.2)	<0.001^*^
24h E	4.0 (3.2,4.7)	5.2 (3.3,26.7)	0.160
24h DA	202.9 (164.0,304.8)	214.5 (149.6,325.4)	0.737

SBP.systolic blood pressure; DBP.diastolic blood pressure; PCC.pheochromocytoma; PGL.paraganglioma;

HN-PGL.head and neck paraganglioma; T-PGL.thoracic paraganglioma; R-PGL.retroperitoneal paraganglioma; P-PGL.pelvic paraganglioma;

NE.Norepinephrine, normal range: 16.7~40.7 μg/24 h; E.Epinephrine, normal range: 1.7~6.4 μg/24 h; DA.Dopamine, normal range: 120.9~330.6 μg/24 h.

*Significant difference between PPGL with somatic mutations in the hypoxia pathway and with somatic mutations in the kinase signaling pathway (P < 0.05).

**Table 5:** Comparison of clinical characteristics of PPGL in the hypoxia pathway.

Clinicalcharacteristics	PPGL with somatic mutations in the Hypoxia pathway (n=14)	PPGL with germline mutations in the Hypoxia pathway (n=21)	P Value
Sex(M/F)	3/11	11/10	0.139
Age(year)	36 (26,50)	30 (23,37)	0.121
Duration(month)	11 (7,24)	48 (12,84)	0.036^*^
SBP	180 (160,198)	280 (160,210)	0.427
DBP	100 (100,110)	110 (110,120)	0.085
Family History	0/14	5/21	0.069
Multiple	2/14	4/21	1.000
Location			
PCC	7/14	4/21	0.073
PGL	8/14	19/21	0.039^*^
HN-PGL	0/14	3/21	0.259
T-PGL	0/14	2/21	0.506
R-PGL	8/14	15/21	0.383
P-PGL	0/14	2/21	0.506
Tumor size(cm)	4.8 (3.0,6.0)	4.0 (2.9,6.0)	0.538
Metastasis	1/14	6/21	0.262
24h NE	258.5 (115.8,1052.1)	250.4 (130.5,746.5)	0.092
24h E	4.0 (3.2,4.7)	3.5 (2.7,4.3)	0.354
24h DA	202.9 (164.0,304.8)	296.1 (206.3,453.6)	0.099

SBP.systolic blood pressure; DBP.diastolic blood pressure; PCC.pheochromocytoma; PGL.paraganglioma;

HN-PGL.head and neck paraganglioma; T-PGL.thoracic paraganglioma; R-PGL.retroperitoneal paraganglioma; P-PGL.pelvic paraganglioma;

NE.Norepinephrine, normal range: 16.7~40.7 μg/24 h; E.Epinephrine, normal range: 1.7~6.4 μg/24 h; DA.Dopamine, normal range: 120.9~330.6 μg/24 h.

*Significant difference between PPGL with somatic mutations in the hypoxia pathway and with germline mutations in the hypoxia pathway (P < 0.05).

### Actionable Gene Mutations in PPGL

Overall, 90 cases of PPGL (84%) exhibited at least one potentially actionable mutation in genes, including *MUC16* (33/107, 31%), *HRAS* (13/107, 12%), *LRP1B* (9/107, 8%), *TCS1* (8/107, 7%), *RET* (7/107, 7%), *TCS2* (7/107, 7%), *KMT2D* (7/107, 7%), *ACO2* (6/107, 6%), and *NTRK1* (6/107, 6%). Only 17 (16%) PPGL cases exhibited no actionable mutations (**Supplementary Figure 2**). Actionable mutations in 13 genes were proved to be linked to targeted therapy in clinical trials in other tumor types (not PPGL). Interestingly, receptor tyrosine kinase (RTK), TK, and mitogen-activated protein kinase (MAPK) pathways were the most frequently involved pathways with a rate of 39% (42/107; **Table 6**).

**Table 6:** Actionable genomic variants in 107 PPGL.

Pathway	Genes	Alterations	Frequency	Target Drugs
Receptor tyrosine kinase	*RET*	C634Y,D631Y C634W,M918T,R418Q,M1008V	7%	Ruxolitinib Vandetanib, Ceritinib, Crizotinib, Everolimus, Lapatinib, Pertuzumab, Gefitinib, Emtansine, Trastuzumab, Ruxolitinib, Sorafenib,Sunitinib, Dasatinib,Nilotinib, Imatinib,Regorafenib
	*ALK*	V349I,S737L, R1231Q,A348T	4%	
	*PDGFRA*	D1033V,T83M	3%	
	*JAK2*	C68R, E890K,L892V	3%	
	*ERBB2*	Q57R,R1146W	2%	
	*FLT3*	G64R,R973X	2%	
	*KIT*	T380M	1%	
Tyrosine kinase	*ABL1*	G706V	2%	Dasatinib,Nilotinib,Ponatinib, Bosutinib,Imatinib
MAPK	*HRAS*	Q61K, Q61R, A121T	12%	Sunitinib,Dasatinib, Vemurafenib,Nilotinib, Dabrafenib,Trametinib, Sorafenib,Imatinib, Regorafenib
	*BRAF*	F595L,K601E	2%	
PI3K-Akt-mTOR	*PIK3CA*	H1047R	1%	Lapatinib,Trastuzumab
Cell cycle	*TP53*	V83fs,P146R	6%	Abemaciclib, Palbociclib Ribociclib, Rituximab Lenalidomide
Frizzled class receptor	*SMO*	G299D, S590T	2%	Vismodegib

## Discussion

We established an expanded panel of 260 genes from known pathogenic pathways associated with PPGL, neuroendocrine tumors, and other cancers to explore the mutation spectrum in PPGL tissues. Our study helped to shed light on the mutation characteristics of PPGL in Chinese population. Furthermore, novel pathogenic mutations in PPGL tissues were observed. Clinical characteristics of PPGL with or without germline mutation and PPGL with mutated genes from various pathways were analyzed. Besides, molecular findings that could possibly serve for targeted therapy were identified.

Mutation profiling was different in several cohorts (**Table 7**). It was worth noting that *SDHx* was more frequently mutated, whereas *FGFR1* was less frequently mutated in our study than the preceding cohort from China (18.7% vs. 4.4%, P < 0.05; 1.9% vs. 9.2%, P < 0.05, respectively) (20). *SDHx* mutations were common in PGL and metastatic PPGL, whereas *FGFR1* mutations were expected in sporadic PGL. The proportion of PGL and metastatic PPGL in cohorts could explain the disparities in mutation frequency. Furthermore, by integrating our results with those of Jiang et al. (**Table 7**), we discovered that the mutation frequency of *EPAS1* was much higher and that of *NF1* was significantly lower in Chinese cohorts than in European cohorts. The different mutation rates of *EPAS1* and *NF1* might arise from racial differences.

**Table 7:** Comprison of gene mutation frequency between different cohorts.

Genes	Our cohortn=107	Chinese cohort in Jiang J et al. study (20)n=697	European cohort (20)n=866
	n (%)	n (%)	n (%)
Mutations identified	66 (61.7)	412 (59.1)	567 (65.5)
*SDHx* ^*^	20 (18.7)	31 (4.4)	108 (12.5)
PCC/PGL	2/18	14/17	27/81
*HRAS*	12 (11.2)	107 (15.4)	68 (7.9)
PCC/PGL^#^	5/7	68/39	63/5
*EPAS1* ^#^	9 (8.4)	41 (5.9)	27 (3.1)
PCC/PGL	2/7	22/19	12/15
*RET*	5 (4.7)	51 (7.3)	91 (10.5)
PCC/PGL^#^	4/1	49/2	91/0
*VHL* ^#^	3 (2.8)	60 (8.6)	107 (12.4)
PCC/PGL	3/0	53/7	93/14
*NF1* ^#^	3 (2.8)	46 (6.6)	133 (15.4)
PCC/PGL^#^	2/1	42/4	129/4
*FGFR1* ^*^	2 (1.9)	64 (9.2)	14 (1.8)
PCC/PGL^#^	0/2	27/37	12/2

PCC.pheochromocytoma; PGL.paraganglioma;

*Significant difference (P < 0.05) in the observational values between our cohort and the study by Jiang J et al. study.

^#^Significant difference (P < 0.05) in the observational values between our cohort and the study by European study.

In addition, some hotspot mutations that were common in other solid tumors were detected in PPGL in our study. These genes were mutually exclusive with germline mutations in disease-causing genes. The hotspot mutation H1047R in oncogenic *PIK3CA* was often detected in breast cancer (21), which enhances the enzymatic activity of PI3K and activates the AKT/mTOR signaling pathway. Hotspot mutation L206R in *PRKACA*, which encodes the catalytic subunit of cyclic-adenosine-monophosphate-dependent protein kinase, was previously reported in adrenocortical adenomas associated with corticotropin-independent Cushing’s syndrome (22). This suggested that the pathogenic mechanism in some common solid tumors should not be ignored in PPGL to provide more possibilities for selecting the treatments for PPGL.

By comparing PPGL cases with mutations in different pathways, we discovered that PPGL with mutations in the hypoxia pathway had an earlier onset age and a higher norepinephrine level at the germline and somatic levels. Indeed, although the kinase signaling pathway were proved to be interconnected with the hypoxia pathway in many cancers (23), PPGL with mutations in the kinase signaling pathway still exhibited different clinical characteristics, such as the tumor location, metastasis, and catecholamine secretion, compared with PPGL with mutations in the hypoxia pathway. Recent studies have reported that mutated genes in the hypoxia pathway reduced the expression of PNMT (24), leading to reduced production of epinephrine, which could account for the lower epinephrine level in PPGL with mutations in the hypoxia pathway.

*TP53* pathogenic mutations only occurred in *SDHB*-mutated metastatic PPGL. Overall, 2 patients with a combination of *SDHB* and *TP53* mutations exhibited local recurrence and distant metastasis after surgical resection. Similarly, several studies detected simultaneous occurrence of *SDHB* and *TP53* mutations in metastatic PPGL (4, 25). In addition, *ATRX* loss of function mutations, which were associated with poor prognosis in PPGL (26, 27), were detected in a case of metastatic, bladder, and abdominal PGLs each. In the follow-up, the outcome (especially recurrence or metastasis) of these patients with PPGL should be closely observed.

In our study, RTK, TK, and MAPK were the most commonly activated pathways in PPGL. These pathways are involved in cell proliferation and transformation and are closely associated with tumorigenesis. Our results indicated that the inhibitors of TK and MAPK could be the most effective targeted therapy for PPGL. At present, targeted therapy for PPGL has been a research hotspot; however, only a few clinical trials or studies have been completed (28–30). Among them, sunitinib was the most intensively studied and reported to have good efficacy. In 2012, Ayala-Ramirez *et al*. retrospectively studied 17 patients with metastatic PPGL treated with sunitinib. Imaging evaluation revealed that 21.4% patients attained partial response and 35.7% had stable disease (31). Several clinical trials are now underway to further confirm the efficacy of sunitinib therapy in patients with progressive PPGL (NCT01371201, NCT00843037). Moreover, phase II clinical trials are underway for axitinib and lenvatinib therapy for PPGL (NCT01967576, NCT03008369 and NCT03839498) (32).

## Conclusion

We detected genetic mutations in 107 cases of PPGL and obtained the specific mutational profile of PPGL at our center. Our results indicated that mutations in pathogenic genes in the common tumors could also be detected in rare PPGL tumors. In addition, PPGL with mutated genes in the hypoxia pathway had earlier onset age and higher level of norepinephrine than PPGL with mutated genes in the kinase signaling pathway. This feature was of great significance at the germline and somatic levels. Finally, new molecular findings that could provide important information for targeted therapy were identified. Mutations in PPGL were mainly concentrated in the RTK, TK, and MAPK pathways, suggesting the potential molecular therapeutic targets in PPGL.

## Data Availability Statement

The data presented in the study are deposited in the Genome Sequence Archive in National Genomics Data Center, Beijing Institute of Genomics (China National Center for Bioinformation), Chinese Academy of Sciences, accession number HRA000415.

## Ethics Statement

The studies involving human participants were reviewed and approved by Ethics Committee of Peking Union Medical College Hospital. Written informed consent to participate in this study was provided by the participants’ legal guardian/next of kin.

## Author Contributions

AT was contributed to the study conception and design. Material preparation, data collection were performed by AT, XM, FW, YC, JW, ZJ, SC and YL. Data analysis was performed by XM, CL, MZ, CZ, and AT. The first draft of the manuscript was written by XM and revised by AT. All authors commented on previous versions of the manuscript. All authors read and approved the final manuscript. All authors contributed to the article and approved the submitted version.

## Funding

This research was funded by CAMS Innovation Fund for Medical Sciences (CIFMS), grant number 2021-I2M-C&T-B-002, 2017-I2M-1-001.

## Conflict of Interest

Authors MZ and CZ were employed by the company Novogene Bioinformatics Technology Co., Ltd.

The remaining authors declare that the research was conducted in the absence of any commercial or financial relationships that could be construed as a potential conflict of interest.

## Publisher’s Note

All claims expressed in this article are solely those of the authors and do not necessarily represent those of their affiliated organizations, or those of the publisher, the editors and the reviewers. Any product that may be evaluated in this article, or claim that may be made by its manufacturer, is not guaranteed or endorsed by the publisher.

## Supplementary Material

Supplementary Figure 1 Study flow of 107 PPGL cases.

Supplementary Figure 2 Actionable mutations in 107 PPGL cases.
