# Supplementary figures and images for: Mutational Profile and Potential Molecular Therapeutic Targets of Pheochromocytoma
Source: Front Endocrinol (Lausanne). 2022 Jul 28;13:921645. doi: 10.3389/fendo.2022.921645 (PMC9368203; doi:10.3389/fendo.2022.921645)

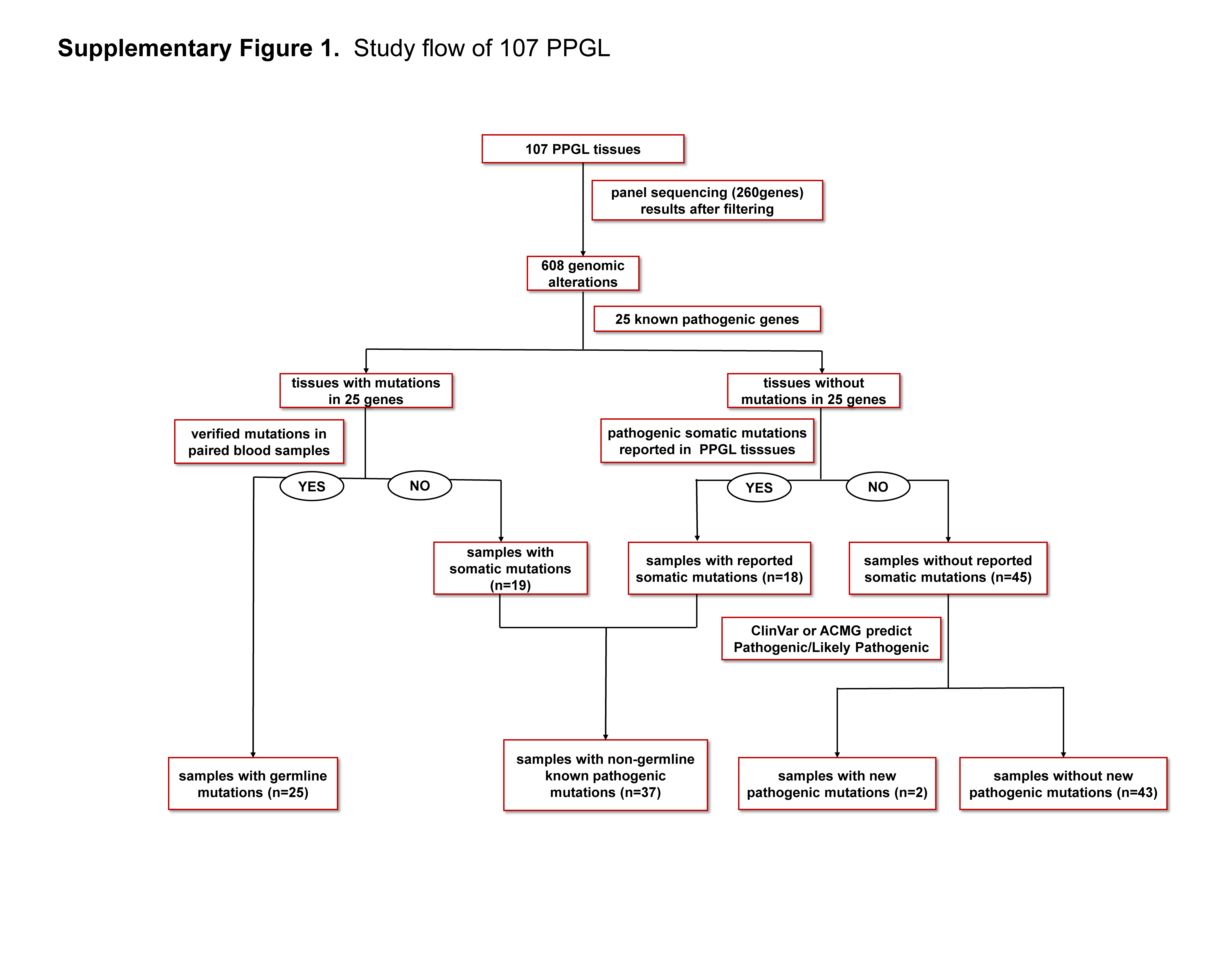

Supplement: Supplementary Figure 1 — Study flow of 107 PPGL cases. [file Image_1.tif]

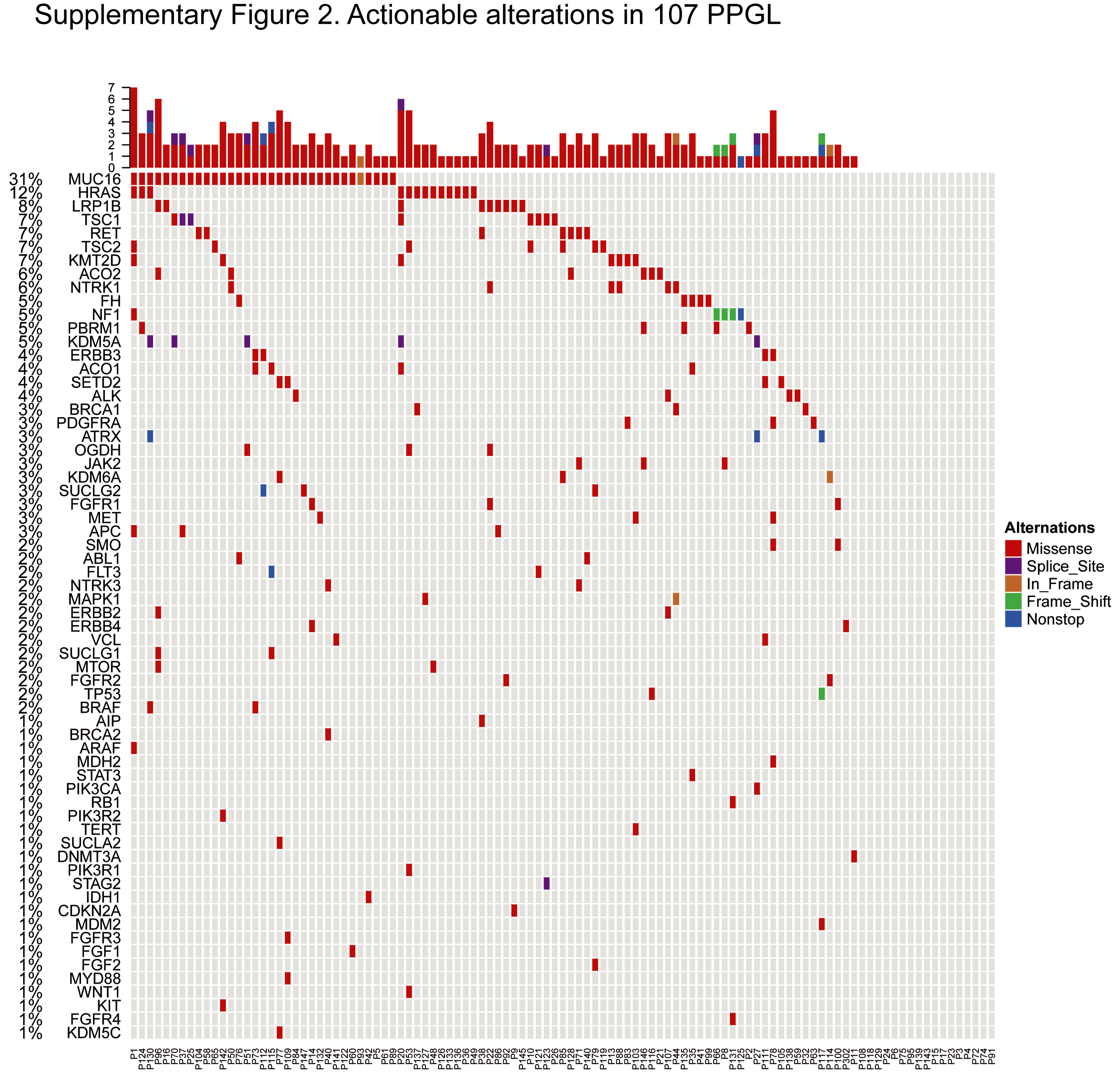

Supplement: Supplementary Figure 2 — Actionable mutations in 107 PPGL cases. [file Image_2.tif]
